# Metagenomics of the midgut microbiome of *Rhipicephalus microplus* from China

**DOI:** 10.1186/s13071-022-05161-6

**Published:** 2022-02-08

**Authors:** Xue-Ling Zhang, Yuan-Ping Deng, Tian Yang, Le-Yan Li, Tian-Yin Cheng, Guo-Hua Liu, De-Yong Duan

**Affiliations:** Research Center for Parasites & Vectors, College of Veterinary Medicine, Hunan Agricultural University, Changsha, 410128 Hunan province China

**Keywords:** *Rhipicephalus microplus*, Metagenomics, Midgut, Microbiome, Gene function

## Abstract

**Background:**

Ticks, which are ectoparasites of animals, may carry multiple pathogens. The cattle tick *Rhipicephalus microplus* is an important bovine parasite in China. However, the midgut microbiome of *R. microplus* from China has not been characterized via metagenomic methods.

**Methods:**

*Rhipicephalus microplus* were collected from cattle in the city of Changsha in Hunan province, China. The DNA of the midgut contents was extracted from fully engorged adult female *R*. *microplus*. A DNA library was constructed and sequenced using an Illumina HiSeq sequencing platform. SOAPdenovo software was used to assemble and analyze the clean data. The latent class analysis algorithm applied to system classification by MEGAN software was used to annotate the information on the species’ sequences. DIAMOND software was used to compare unigenes with the Kyoto Encyclopedia of Genes and Genomes (KEGG) database, and functional annotation was carried out based on the results of the comparison.

**Results:**

The dominant phyla in the five samples were Firmicutes, Proteobacteria, and Actinobacteria. *Streptococcus*, *Mycobacterium*, *Anaplasma*, *Enterococcus*, *Shigella*, *Lactobacillus*, *Brachyspira*, *Pseudomonas*, *Enterobacter*, *Bacillus*, and *Lactococcus* were the dominant genera in the five samples. The endosymbiotic bacterium *Wolbachia* was also detected in all of the samples*. Mycobacterium malmesburyense*, *Streptococcus pneumoniae*, *Anaplasma phagocytophilum*, *Enterococcus faecium*, *Shigella sonnei*, *Enterococcus faecalis*, *Lactobacillus casei*, *Brachyspira hampsonii*, *Pseudomonas syringae*, *Enterobacter cloacae*, and *Lactococcus garvieae* were the dominant species in the five samples. In addition to these bacterial species, we also detected some eukaryotes, such as *Rhizophagus irregularis*, *Enterospora canceri*, *Smittium culicis*, *Zancudomyces culisetae*, *Trachipleistophora hominis*, and viruses such as orf virus, human endogenous retrovirus type W, enzootic nasal tumor virus of goats, bovine retrovirus CH15, and galidia endogenous retrovirus in all of the samples at the species level. The results of the annotated KEGG pathway predictions for the gene functions of the midgut microflora of *R*. *microplus* indicated genes involved in lipid and amino acid metabolism, infectious diseases (e.g., *Streptococcus* *pneumonia* infection, human granulocytic anaplasmosis, *Shigella* *sonnei* infection, *Salmonella enterica* infection, and pathogenic *Escherichia coli* infection), and cancer.

**Conclusions:**

Our study revealed that the midgut microbiome of *R. microplus* is not only composed of a large number of bacteria, but that a portion also comprises eukaryotes and viruses. The data presented here enhance our understanding of this tick’s midgut microbiome and provide fundamental information for the control of ticks and tick-borne diseases.

**Graphical Abstract:**

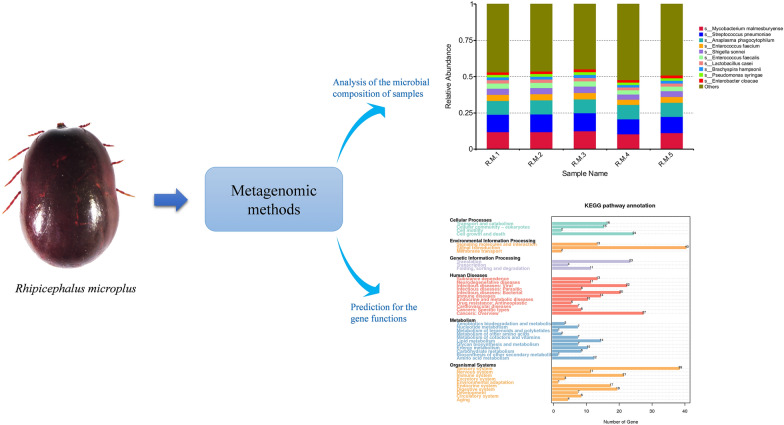

**Supplementary Information:**

The online version contains supplementary material available at 10.1186/s13071-022-05161-6.

## Background

The tick species *Rhipicephalus microplus* belongs to the family Ixodidae. *Rhipicephalus microplus* is widely distributed throughout the world, and epidemics of the diseases for which it is a vector, such as anaplasmosis and babesiosis, are often reported in Brazil, India, tropical and subtropical Asia, the Caribbean, Central and South America, China, and Mexico [1–5]. *Rhipicephalus microplus* is a single-host tick that infects cattle and buffaloes. Although *R*. *microplus* only infests cattle once at each stage of development (larva, nymph, and adult), it feeds on blood for several days at a time. This tick harms its hosts by biting and sucking blood, which lead to pruritus, emaciation, loss of fur quality, anemia, reduced milk production, and other clinical characteristics in infested animals [6].

*Rhipicephalus microplus* is not only a blood-sucking parasite but also the vector for a variety of pathogens [7, 8]. It is considered to be the most important vector of bovine tick-borne diseases in global agroecosystems [9, 10]. Among the pathogens that *R*. *microplus* can carry, we can cite *Anaplasma phagocytophilum*, *Anaplasma marginale*, *Babesia bigemina*, *Babesia bovis*, *Ehrlichia chaffeensis*, *Ehrlichia canis*, *Ehrlichia* sp. Tibet, *Rickettsia* spp*.*, *Borrelia* spp., and severe fever with thrombocytopenia syndrome virus [11–14]. Furthermore, some researchers have detected a novel human pathogen, *Anaplasma capra*, in *R. microplus* [15]. Among the pathogens carried by this tick, *B*. *bigemina*, *B*. *bovis*, and *A*. *marginale* threaten the health of cattle and cause economic losses to the cattle industry [16, 17]; *A*. *phagocytophilum* can cause human granulocytic anaplasmosis, which can be a serious threat to human health [18–20].

Previously, the identification of microorganisms generally depended on culturing. With the rapid development of sequencing technology, bacterial communities associated with entire ticks, the midgut, and the ovary of *R*. *microplus* have been studied using non-culture methods [9]. The dominant bacteria encountered in these studies of *R*. *microplus* were *Wolbachia*, *Coxiella*, and *Borrelia burgdorferi* [9]. Polymerase chain reaction (PCR) combined with denaturing gradient gel electrophoresis, established by Fisher and Lerman in 1983 [21], was first applied to the analysis of microbial populations in 1993 [22], and has been extensively used for the direct identification of the microflora of ticks [23–25]. The bacterial community of the midgut of *R*. *microplus* collected from cattle in Jiangxi and Hunan provinces of China was analyzed by PCR combined with denaturing gradient gel electrophoresis, and the dominant bacteria were found to be *Rickettsia peacockii* and *Coxiella* [25]. In recent years, new ideas and approaches for the study of the gut microbiome of ticks have been developed due to the rise of metagenomics [26, 27].

Metagenomics can be used to identify new and emerging human pathogens circulating in tick vectors [20, 28, 29]. Adegoke et al. [30] analyzed the microbial composition of two tick species (*Hyalomma anatolicum* and *R*. *microplus*) in Pakistan using metagenomic sequencing. In the present study, metagenomics was used to analyze and determine the species of the midgut microbiome of five fully engorged adult female *R*. *microplus* collected from cattle in the city of Changsha in Hunan province, China. Furthermore, the Kyoto Encyclopedia of Genes and Genomes (KEGG) was used to predict the gene functions of the midgut microflora of *R*. *microplus*.

## Methods

### Sample collection and DNA extraction

Twenty fully engorged adult female *R. microplus* were collected from the body surfaces of cattle located in the city of Changsha in Hunan province (28º12′N, 112º59′E), China. All of the *R. microplus* samples were immediately transferred to Hunan Agricultural University. Five fully engorged adult female *R*. *microplus* were utilized for the analysis. Before dissection, the five ticks were surface disinfected with 70% (volume/volume) ethanol for 60 s followed by immersion in three reagents, 100% ethanol, 10% sodium hypochlorite solution, and distilled water, to remove the disinfectant. All of the dissecting apparatuses, plasticware, glassware, buffers (including phosphate-buffered saline), and solutions were sterilized by autoclaving and UV treatment. All of the procedures were conducted in a biosafety cabinet after UV sterilization to protect the samples from environmental contamination.

The five ticks were stabilized with fine-tipped forceps by holding the rear portion. The rear of each tick was cut using sterile ophthalmic scissors, and the midgut contents from each tick (100 μl) were pooled into a single tube. Then 1000 μl of hexadecyltrimethylammonium bromide lysate and 20 μl lysozyme were added to each tube, and the five tubes labeled as follows: R.M.1, R.M.2, R.M.3, R.M.4, and R.M.5. The five tubes were placed in a water bath at 65 °C for 2 h, during which time the tubes were inverted several times to ensure that the samples were fully lysed. After centrifugation (5022 *g* for 10 min), 950 μl supernatant of each sample was absorbed, and the same volume of phenol (pH 8.0):chloroform:isoamyl alcohol (25:24:1) mixture was added and mixed. After centrifugation (8609 *g* for 10 min), the supernatants were absorbed, and the same volume of chloroform:isoamyl alcohol (24:1) was added and mixed. After centrifugation (8609 *g* for 10 min), the supernatant of each sample was transferred to a 1.5-ml centrifuge tube. Then, a 3/4 volume of isopropanol was added to the supernatant of each sample; the tube was shaken and then the sample precipitated at − 20 °C. The sample tubes were centrifuged at 8609 *g* for 10 min, the liquid removed, and the sample washed twice with 1 ml of 75% (volume/volume) ethanol, after which the ethanol was removed. After that, the precipitate of each sample was allowed to dry naturally at room temperature, and then 50 μl double distilled H_2_O was added to dissolve the DNA. The sample was incubated at 60 °C for 10 min, and 1 μl RNase A was added to digest RNA. The sample was then incubated at 37 °C for 15 min.

### Library construction and sequencing

A total of 1 μg DNA per sample was used as input material for the DNA sample preparation. Sequencing libraries were generated using a NEBNext Ultra DNA Library Prep Kit (New England Biolabs, Ipswich, MA) following the manufacturer’s protocol. Briefly, the DNA samples were fragmented by sonication to a size of 350 base pairs (bp), after which the DNA fragments were end-polished, A-tailed, and ligated with the full-length adaptor for Illumina sequencing with further PCR amplification. Finally, the PCR products were purified by the AMPure XP system (Beckman Coulter, Brea, CA). The libraries were analyzed for size distribution using an Agilent 2100 Bioanalyzer (Agilent Technologies, Palo Alto, CA) and quantified using real-time PCR (Thermo Fisher Scientific, Waltham, MA). The clustering of the index-coded samples was performed on a cBot Cluster Generation System (Illumina, San Diego, CA) according to the manufacturer’s instructions. After cluster generation, the library preparations were sequenced on an Illumina HiSeq platform (Illumina), and paired-end reads were generated.

### Pretreatment of sequencing results

Preprocessing of the raw data obtained from the Illumina HiSeq platform (Illumina) using Readfq (v8; https://github.com/cjfields/readfq) was conducted to acquire the clean data for subsequent analysis. The specific processing steps were as follows: removal of the reads that contained low-quality bases (default quality threshold value ≤ 38) above a certain portion (default length of 40 bp); removal of the reads in which the N base had reached a certain percentage (default length of 10 bp); removal of the reads that shared an overlap above a certain portion with the adapter (default length of 15 bp); the reads that were of host origin were also filtered by Bowtie 2.2.4 software (http://bowtiebio.sourceforge.net/bowtie2/index.shtml).

### Metagenome assembly

The clean data were assembled and analyzed using SOAPdenovo software (v2.04; http://soap.genomics.org.cn/soapdenovo.html) [31]. For a single sample,* k*-mer = 55 was selected for assembly to obtain scaffolds of the sample, with the following parameters: -*d* 1, -*M* 3, -*R*, -u, -*F*, -*K* 55 [32–35]. Then, the assembled scaffolds were broken from the N connections to obtain scaftigs without N [32, 36, 37]. All the clean data for the samples were compared to the respective scaftigs using Bowtie 2.2.4 software to acquire the paired-end reads not used. The parameters were as follows: –end-to-end, –sensitive, -*I* 200, -*X* 400 [32]. All reads not used in the forward step for all the samples were combined. Then, SOAPdenovo software (v2.04; http://soap.genomics.org.cn/soapdenovo.html) was used for mixed assembly with the same parameters as for the single assembly. The mixed assembly scaffolds were broken from the N connection to obtain the scaftigs. Any fragments shorter than 500 bp in the scaftigs were filtered. The filtered scaftigs from the single or mixed assembly were statistically analyzed.

### Gene prediction and abundance analysis

MetaGeneMark software (v2.10; http://topaz.gatech.edu/GeneMark/) was used to predict the open reading frame of the scaftigs (≥ 500 bp) from the single and mixed assembly. Sequence information from the predicted results with lengths less than 100 nucleotides [32, 37–40] was filtered. CD-HIT software (v4.5.8; http://www.bioinformatics.org/cd-hit) [41, 42] was used to remove redundancy and obtain the unique initial gene catalogue (the parameter options were –*c* 0.95, –*G* 0, –a*S* 0.9, –*g* 1, –*d* 0 [39, 43]). The longest sequences were selected as the representative sequences, and those sequences with 95% identity and 90% coverage were clustered. The clean data of each sample were mapped to an initial gene catalogue using Bowtie 2.2.4 (the parameter settings were –end-to-end, –sensitive, –*I* 200, –*X* 400 [32, 40]) to obtain the number of reads to which genes mapped in each sample. The genes for which the number of reads was ≤ 2 [40, 44] were filtered in each sample to obtain the gene catalogue (unigenes). The abundance of information for each gene in each sample was calculated based on the number of mapped reads and the length of the gene [38, 39, 45–47]. The basic informative statistics, core-pan gene analysis, correlation analysis of samples, and Venn diagram analysis of the numbers of genes were carried out based on the abundance of each gene in each sample of unigenes.

### Species annotation

DIAMOND software (v0.9.9; https://github.com/bbuchfink/diamond/) [48] was used to compare the unigenes with the sequences of bacteria, fungi, archaea, and viruses that were extracted from the nr database (v2018-01–02; https://www.ncbi.nlm.nih.gov/) of the National Center for Biotechnology Information (NCBI) [the parameter settings were Basic Local Alignment Search Tool Program (BLASTP) E value ≤ 1e−5]. The previous alignment results were employed for the latent class analysis algorithm [49] that was applied to the classification from MEGAN software [50] to verify the species annotation information of the sequences. A table containing the number of genes and the abundance information for each sample in a taxonomic hierarchy (kingdom, phylum, class, order, family, genus, and species) was obtained based on the latent class analysis annotation results and the gene abundance table. The abundance of a species in one sample was equal to the sum of the gene abundances annotated for the species [38, 45, 51]; the number of genes of a species in a sample was equal to the number of genes with nonzero abundance. Krona analysis, the generation of relative abundance, and the construction of an abundance cluster heat map were carried out based on the abundance table of each taxonomic hierarchy.

### Common functional database annotations

DIAMOND software (v0.9.9) was used to compare unigenes with the KEGG [52, 53] database (v2018-01-01; http://www.kegg.jp/kegg/) with the parameter setting of BLASTP, E value ≤ 1e−5 [33, 40]. For each sequence’s alignment result, the best BLAST hit (one high-scoring segment pair > 60 bits) was used for subsequent analysis [33, 40, 54]. The relative abundances of different functional hierarchies were calculated (the relative abundance of each functional level was equal to the sum of the relative abundances of genes annotated at the functional level) [32, 39]. The table of the number of genes of each sample in each taxonomic hierarchy was based on the results of the functional annotation and the table of gene abundances. The number of genes of a function in a sample was equal to the number of genes that was annotated for this function given that the abundance was nonzero. Based on the abundance table of each taxonomic hierarchy, the number of annotated genes was determined; the general relative abundance and the abundance cluster heat map were constructed, and the metabolic pathways were analyzed.

## Results

### Test results of DNA quality

A total of 26,678.61 Mbp of clean data were generated by sequencing with the Illumina HiSeq platform. The effective data rate was 99.7%. Specific data output statistics and quality control information are shown in Table 1.

**Table 1:** Quality control results for metagenomic DNA of each sample

Sample^a^	InsertSize^b^ (bp)	RawData^c^	CleanData^d^	Clean Q20^e^	Clean Q30^f^	Clean GC^g^ (%)	Effective^h^ (%)
R.M.1	350	5129.63	5109.71	97.43	93.02	44.62	99.61
R.M.2	350	5487.17	5472.01	97.63	93.44	44.58	99.72
R.M.3	350	5240.75	5226.79	97.77	93.79	44.95	99.73
R.M.4	350	5499.14	5481.69	97.72	93.77	45.28	99.68
R.M.5	350	5402.47	5388.41	97.72	93.72	45.11	99.74

^a^Sample name

^b^Use of the 350-base pair library

^c^Raw data off the computer

^d^Effective data obtained by filtering

^e^Sequencing error rate in CleanData is < 0.01 (quality is the percentage of bases with a value > 20)

^f^Sequencing error rate in CleanData is < 0.001 (quality is the percentage of bases with a value > 30)

^g^GC proportion of the bases in CleanData

^h^Percentage of valid data (CleanData) and raw data (RawData)

### General statistics

**Fig. 1:**
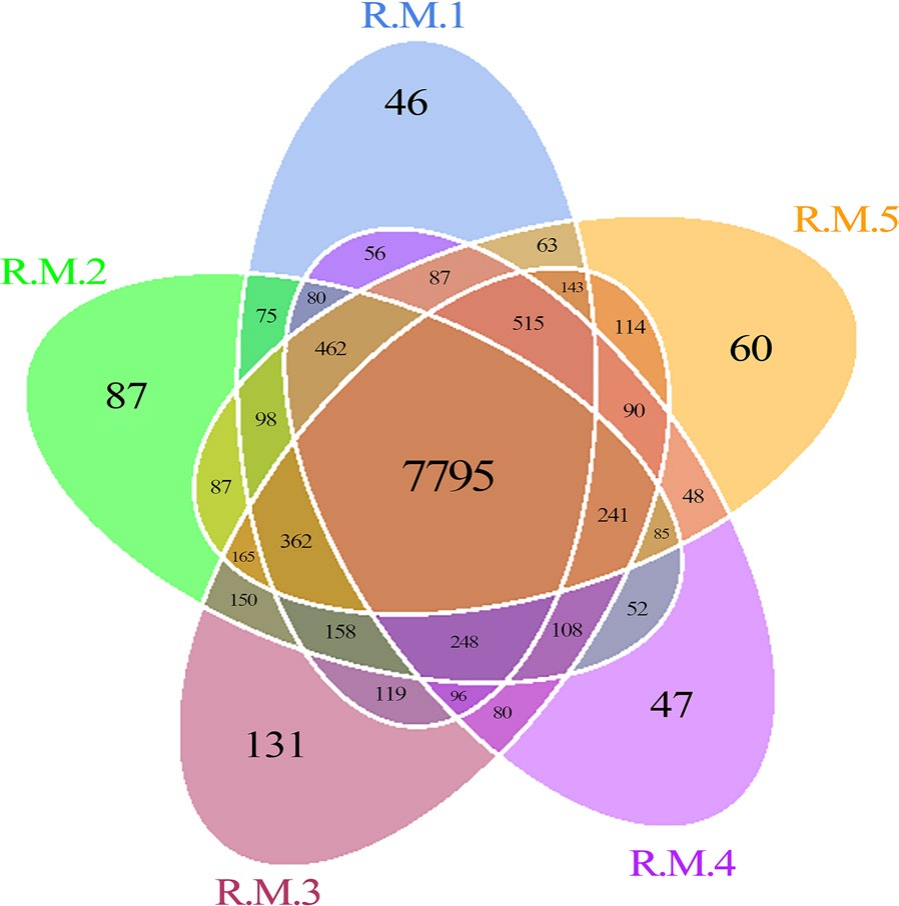
Venn diagram analysis of the number of genes of the samples of *Rhipicephalus microplus*.* Each circle* represents a sample; the numbers in the* overlapping areas* indicate the number of genes shared between the samples. The numbers in the* non-overlapping areas* indicate the number of unique genes in the sample

Following quality control, 10,403, 10,253, 10,515, 10,090, and 10,415 genes were obtained from the sequencing data of R.M.1, R.M.2, R.M.3, R.M.4, and R.M.5, respectively. The distribution of the genes of the five samples is shown in Fig. 1. The number of genes common to all five samples was 7,795. The numbers of genes specific to R.M.1, R.M.2, R.M.3, R.M.4, and R.M.5 were 46, 87, 131, 47, and 60, respectively. These results suggest that a large proportion of the microbial population of the five samples was identical, and that relatively small differences existed between individuals.

### Relative abundance of microorganisms

**Fig. 2:**
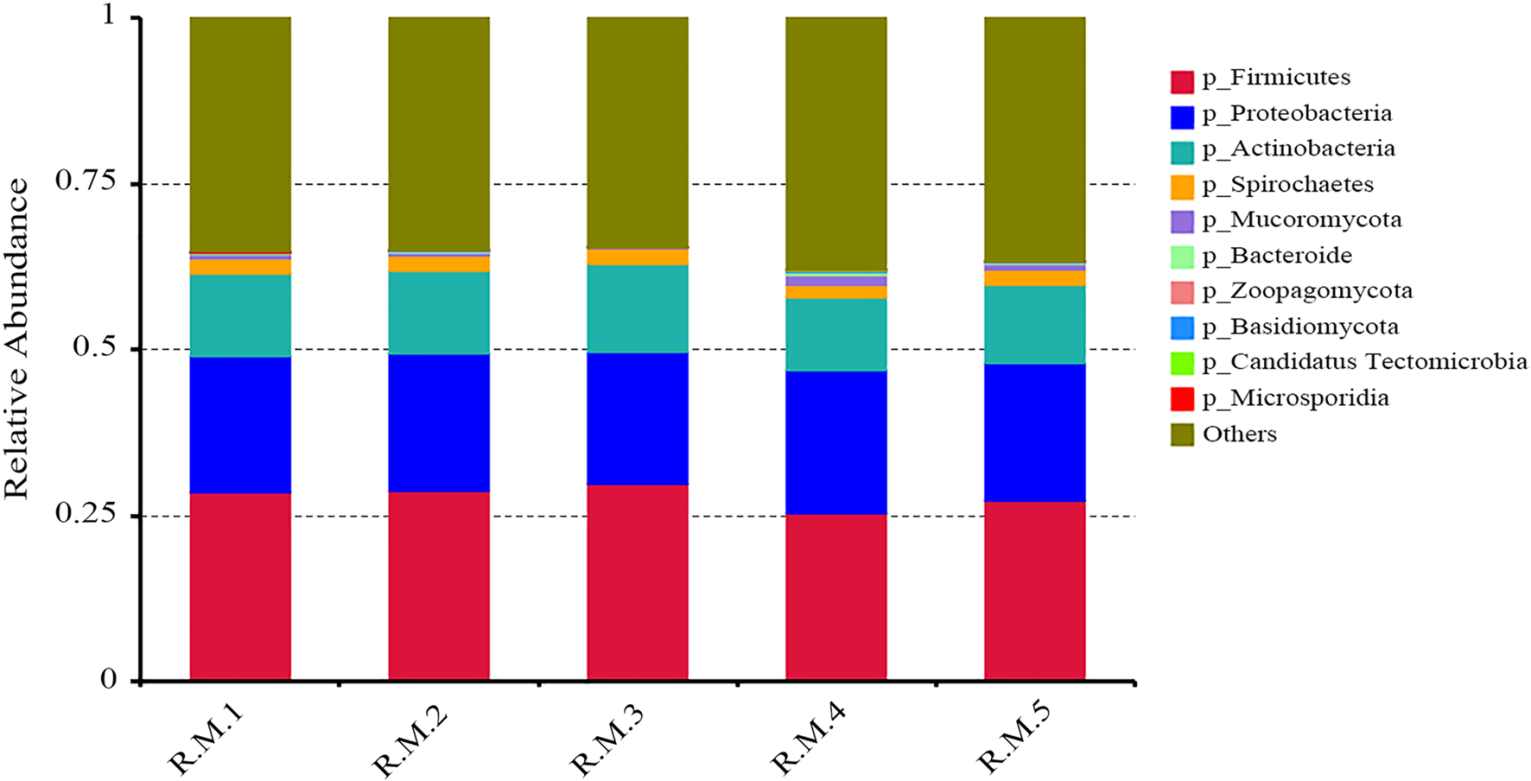
Microbial population characteristics of the 10 most abundant phyla in the five samples of *Rhipicephalus microplus*

Sixteen phyla were common to the five samples. The microbial population characteristics of the 10 most abundant phyla in the five samples are shown in Fig. 2. Of these, Firmicutes, Proteobacteria, Actinobacteria, and Spirochaetes were the main phyla in all the samples. Firmicutes was predominant (≥ 25% of genes) in all samples.

**Fig. 3:**
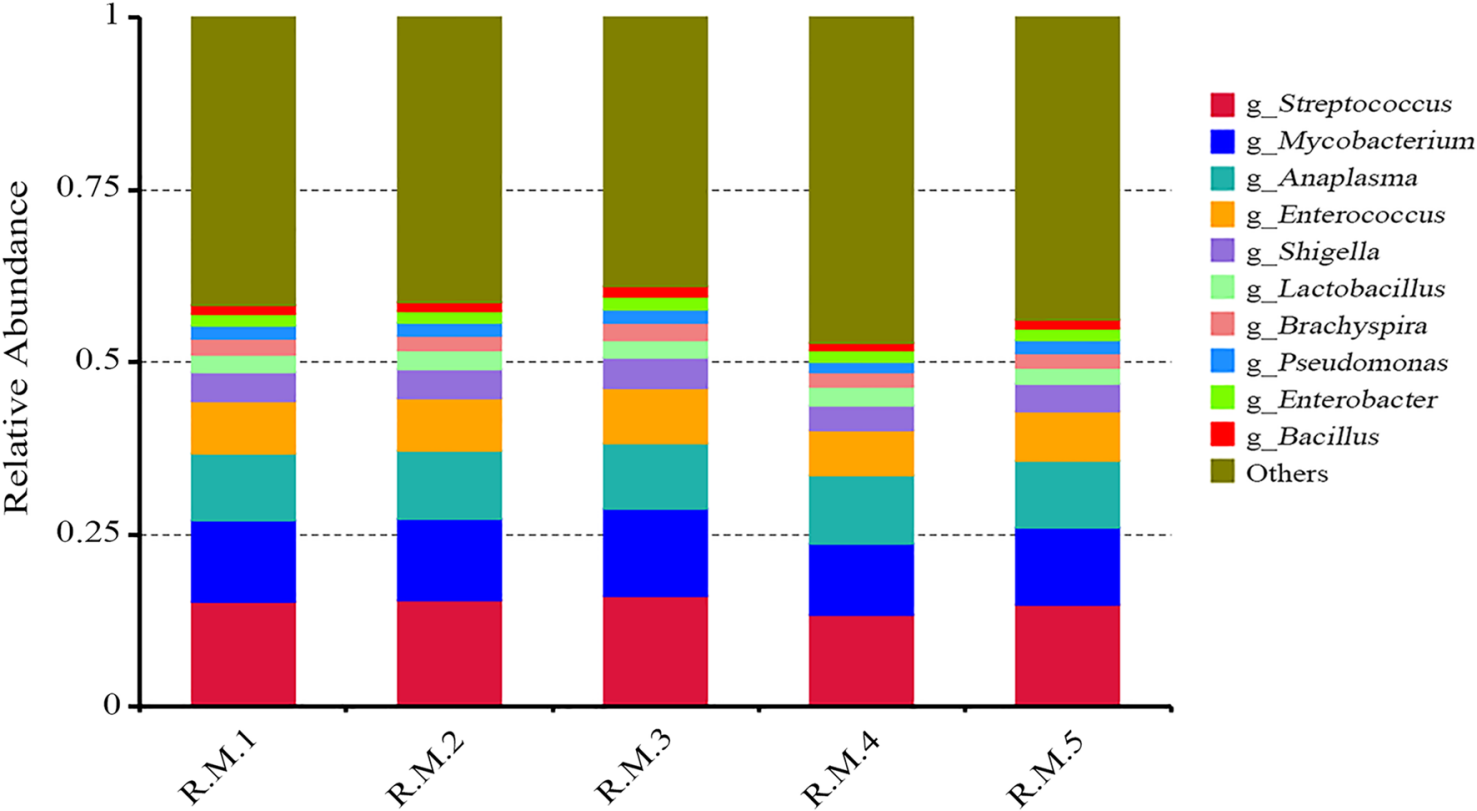
Microbial population characteristics of the 10 most abundant genera in the five samples of *Rhipicephalus microplus*

Ninety-five genera were common to the five samples. The relative abundances of these 95 genera are shown in Additional file 1: Table S1. Of these, *Streptococcus*, *Mycobacterium*, *Anaplasma*, *Enterococcus*, *Shigella*, *Lactobacillus*, *Brachyspira*, *Pseudomonas*, *Enterobacter*, *Bacillus*, and *Lactococcus* were the dominant genera in the five samples (Fig. 3). Five genera (*Mycobacterium*, *Brachyspira*, *Campylobacter*, *Occidentia*, and *Neisseria*) that were not reported in previous studies of *R*. *microplus* were thus, to the best of our knowledge, found for the first time in this species in the present study, despite their abundances being relatively low. *Coxiella* was not found in the midgut of *R*. *microplus* in the present study.

At the species level, 144 species were common to the five samples. These included 76 bacterial species, 47 eukaryotes, and 21 viral species. The relative abundances of the most common 35 bacterial species, the 47 eukaryotes, the 21 viral species, and other bacterial species are shown in Tables 2, 3, 4 and Additional file 1: Table S2, respectively. Of the bacterial species, *Mycobacterium malmesburyense*, *Streptococcus pneumoniae*, *Anaplasma phagocytophilum*, *Enterococcus faecium*, *Shigella sonnei*, *Enterococcus faecalis*, *Lactobacillus casei*, *Brachyspira hampsonii*, *Pseudomonas syringae*, *Enterobacter cloacae*, and *Lactococcus garvieae* were the dominant ones in the five samples. The dominant eukaryotes were *Rhizophagus irregularis*, *Enterospora canceri*, *Smittium culicis*, *Zancudomyces culisetae*, and *Trachipleistophora hominis*. Orf virus, human endogenous retrovirus (HERV) type W (HERV-W), enzootic nasal tumor virus of goats, bovine retrovirus CH15, and galidia endogenous retrovirus were the dominant viruses.

**Table 2:** Relative abundance of the 35 most common bacterial species of the five samples of *Rhipicephalus microplus*

Bacterial species	Abundance				
	R.M.1	R.M.2	R.M.3	R.M.4	R.M.5
*Streptococcus pneumoniae*	0.1196310	0.1215607	0.1235076	0.1039026	0.1119861
*Mycobacterium malmesburyense*	0.1181283	0.1184928	0.1255925	0.1030581	0.1127683
*Anaplasma phagocytophilum*	0.0969749	0.0982932	0.0960091	0.0984723	0.0972878
*Enterococcus faecium*	0.0416463	0.0417757	0.0443746	0.0365036	0.0396334
*Shigella sonnei*	0.0427119	0.0430214	0.0437496	0.0366385	0.0399201
*Enterococcus faecalis*	0.0343390	0.0344909	0.0351445	0.0294956	0.0319857
*Lactobacillus casei*	0.0231117	0.0234679	0.0229566	0.0194934	0.0209869
*Brachyspira hampsonii*	0.0193290	0.0188721	0.0213881	0.0169518	0.0187922
*Pseudomonas syringae*	0.0183325	0.0185135	0.0198217	0.0162424	0.0176270
*Enterobacter cloacae*	0.0168716	0.0175321	0.0193554	0.0162633	0.0177204
*Lactococcus garvieae*	0.0125082	0.0123325	0.0126887	0.0109034	0.0121182
*Solemya velum* gill symbiont	0.0050665	0.0044121	0.0010743	0.0113297	0.0069088
*Bacillus obstructivus*	0.0093129	0.0092661	0.0101419	0.0082177	0.0090449
*Campylobacter jejuni*	0.0084139	0.0085832	0.0083415	0.0073301	0.0079475
*Rickettsia* endosymbiont	0.0032815	0.0028788	0.0006970	0.0073949	0.0044852
*Lactobacillus plantarum*	0.0013561	0.0016254	0.0014914	0.0068720	0.0014044
*Corynebacterium diphtheriae*	0.0059176	0.0065282	0.0060945	0.0052200	0.0055256
*Escherichia coli*	0.0030110	0.0026547	0.0008087	0.0064927	0.0039222
*Neisseria polysaccharea*	0.0062392	0.0064022	0.0061740	0.0052582	0.0056720
*Ehrlichia minasensis*	0.0019209	0.0016806	0.0003970	0.0043562	0.0025865
*Brachyspira hyodysenteriae*	0.0031406	0.0032647	0.0032565	0.0027490	0.0030561
*Candidatus* Nephrothrix sp. EaCA	0.0013131	0.0011206	0.0002492	0.0028623	0.0017245
*Bacillus* sp. VT-16–64	0.0017523	0.0017518	0.0018561	0.0014193	0.0016021
*Eggerthia catenaformis*	0.0015535	0.0015911	0.0016787	0.0013054	0.0014979
*Bacillus cereus*	0.0013887	0.0011124	0.0015785	0.0009201	0.0014289
Bacterium 2013Ark19i	0.0006967	0.0006075	0.0001450	0.0015662	0.0009566
*Lactobacillus rhamnosus*	0.0012846	0.0012983	0.0014517	0.0012424	0.0012509
*Clostridioides difficile*	0.0011013	0.0011726	0.0012640	0.0010050	0.0010989
*Candidatus* Entotheonella sp. TSY2	0.0005071	0.0004457	0.0001062	0.0011905	0.0006818
*Wolbachia* endosymbiont	0.0008269	0.0007424	0.0001854	0.0019281	0.0011074
*Solemya pervernicosa* gill symbiont	0.0004455	0.0004001	0.0000882	0.0010461	0.0006319
*Rickettsia amblyommatis*	0.0004077	0.0003603	0.0000835	0.0009656	0.0005706
*Flavobacterium* sp. JRM	0.0001648	0.0001505	0.0000356	0.0004114	0.0002374
*Epulopiscium* sp. SCG-C07WGA-EpuloA2	0.0001750	0.0001522	0.0000373	0.0003862	0.0002485

**Table 3:** Relative abundance of eukaryotes at the species level of the five samples of *Rhipicephalus microplus*

Species	Abundance				
	R.M.1	R.M.2	R.M.3	R.M.4	R.M.5
*Rhizophagus irregularis*	0.0061110	0.0054017	0.0013369	0.0137931	0.0082716
*Enterospora canceri*	0.0003404	0.0002874	0.0000645	0.0007332	0.0004577
*Smittium culicis*	0.0003163	0.0002714	0.0000632	0.0007328	0.0004257
*Zancudomyces culisetae*	0.0002985	0.0002536	0.0000634	0.0006993	0.0004066
*Trachipleistophora hominis*	0.0001442	0.0001312	0.0000301	0.0003238	0.0002033
*Armillaria ostoyae*	0.0000963	0.0000870	0.0000188	0.0002133	0.0001240
*Sporothrix schenckii*	0.0000951	0.0000759	0.0000184	0.0002106	0.0001198
*Puccinia striiformis*	0.0000909	0.0000690	0.0000144	0.0001798	0.0001031
*Trametes cinnabarina*	0.0000616	0.0000710	0.0000138	0.0001678	0.0000895
*Lichtheimia corymbifera*	0.0000771	0.0000660	0.0000156	0.0001624	0.0000990
*Rhizopus microsporus*	0.0000597	0.0000586	0.0000117	0.0001448	0.0000860
*Trametes pubescens*	0.0000564	0.0000564	0.0000137	0.0001386	0.0000843
*Rhizopus delemar*	0.0000475	0.0000449	0.0000093	0.0001180	0.0000647
*Rhizoctonia solani*	0.0000371	0.0000317	0.0000071	0.0000882	0.0000512
*Nosema apis*	0.0000298	0.0000224	0.0000062	0.0000686	0.0000319
*Ceraceosorus bombacis*	0.0000262	0.0000209	0.0000060	0.0000659	0.0000335
*Erysiphe necator*	0.0000342	0.0000254	0.0000061	0.0000657	0.0000417
*Umbilicaria pustulata*	0.0000200	0.0000177	0.0000040	0.0000524	0.0000227
*Smittium mucronatum*	0.0000173	0.0000194	0.0000044	0.0000488	0.0000253
*Trichosporon asahii*	0.0000175	0.0000181	0.0000026	0.0000464	0.0000277
*Phycomyces blakesleeanus*	0.0000290	0.0000255	0.0000048	0.0000458	0.0000330
*Candida albicans*	0.0000204	0.0000188	0.0000053	0.0000453	0.0000274
*Lichtheimia ramosa*	0.0000221	0.0000150	0.0000039	0.0000447	0.0000283
*Candida glabrata*	0.0000153	0.0000119	0.0000018	0.0000394	0.0000206
*Mucor circinelloides*	0.0000168	0.0000111	0.0000033	0.0000364	0.0000206
*Tuber aestivum*	0.0000137	0.0000094	0.0000035	0.0000349	0.0000193
*Fusarium langsethiae*	0.0000146	0.0000132	0.0000037	0.0000345	0.0000181
*Penicillium subrubescens*	0.0000162	0.0000120	0.0000030	0.0000331	0.0000193
*Microbotryum intermedium*	0.0000146	0.0000139	0.0000027	0.0000327	0.0000173
*Choanephora cucurbitarum*	0.0000117	0.0000126	0.0000022	0.0000308	0.0000176
*Mycena chlorophos*	0.0000149	0.0000121	0.0000040	0.0000301	0.0000217
*Ganoderma sinense*	0.0000117	0.0000086	0.0000030	0.0000268	0.0000169
*Chaetomium globosum*	0.0000124	0.0000115	0.0000026	0.0000259	0.0000189
*Pochonia chlamydosporia*	0.0000099	0.0000082	0.0000020	0.0000243	0.0000132
*Kazachstania exigua*	0.0000101	0.0000078	0.0000015	0.0000231	0.0000106
*Hypholoma sublateritium*	0.0000111	0.0000081	0.0000017	0.0000221	0.0000112
*Aspergillus oryzae*	0.0000091	0.0000102	0.0000021	0.0000219	0.0000137
*Aspergillus cristatus*	0.0000069	0.0000085	0.0000019	0.0000185	0.0000115
*Macrophomina phaseolina*	0.0000076	0.0000053	0.0000024	0.0000163	0.0000079
*Nosema ceranae*	0.0000048	0.0000049	0.0000010	0.0000113	0.0000040
*Sphaerobolus stellatus*	0.0000071	0.0000064	0.0000017	0.0000047	0.0000081
*Gonapodya prolifera*	0.0000032	0.0000046	0.0000011	0.0000077	0.0000037
*Basidiobolus meristosporus*	0.0000067	0.0000028	0.0000055	0.0000020	0.0000051
*Syncephalastrum racemosum*	0.0000059	0.0000034	0.0000048	0.0000022	0.0000048
*Tilletia indica*	0.0000027	0.0000031	0.0000002	0.0000047	0.0000026
*Penicillium antarcticum*	0.0000018	0.0000047	0.0000006	0.0000030	0.0000019
*Aspergillus calidoustus*	0.0000021	0.0000013	0.0000004	0.0000015	0.0000016

**Table 4:** Relative abundance of the 21 viral species of the five samples of *Rhipicephalus microplus*

Viral species	Abundance				
	R.M.1	R.M.2	R.M.3	R.M.4	R.M.5
*Orf virus*	0.0085001	0.0082064	0.0090526	0.0074134	0.0081414
*Human endogenous retrovirus W*	0.0007523	0.0007795	0.0008286	0.0006669	0.0007232
*Enzootic nasal tumor virus* of goats	0.0001849	0.0002783	0.0002061	0.0001104	0.0001730
*Bovine retrovirus CH15*	0.0002075	0.0002368	0.0002332	0.0002082	0.0002009
*Galidia ERV*	0.0001104	0.0001267	0.0001032	0.0000943	0.0001037
*Cotesia sesamiae bracovirus*	0.0000526	0.0000554	0.0000101	0.0001018	0.0000655
*Chelonid alphaherpesvirus 5*	0.0000740	0.0000944	0.0000974	0.0000782	0.0000781
*Human endogenous retrovirus*	0.0000718	0.0000876	0.0000912	0.0000663	0.0000762
*Human endogenous retrovirus K*	0.0000664	0.0000747	0.0000639	0.0000520	0.0000528
*Mouse mammary tumor virus*	0.0000631	0.0000615	0.0000585	0.0000502	0.0000501
*Bat gammaretrovirus*	0.0000250	0.0000298	0.0000291	0.0000256	0.0000333
*Lymphocystis disease virus*—isolate China	0.0000168	0.0000115	0.0000027	0.0000275	0.0000180
*Simian retrovirus Y*	0.0000071	0.0000118	0.0000115	0.0000079	0.0000085
*Lymphocystis disease virus Sa*	0.0000077	0.0000036	0.0000008	0.0000115	0.0000060
*Elephant endotheliotropic herpesvirus 4*	0.0000098	0.0000101	0.0000106	0.0000080	0.0000076
*Bovine endogenous retrovirus beta 1*	0.0000065	0.0000066	0.0000083	0.0000034	0.0000058
*Squirrel monkey retrovirus*	0.0000040	0.0000072	0.0000071	0.0000047	0.0000038
*Murine leukemia virus*	0.0000060	0.0000066	0.0000069	0.0000050	0.0000046
*Feline leukemia virus*	0.0000058	0.0000043	0.0000037	0.0000052	0.0000059
*Human endogenous retrovirus H*	0.0000018	0.0000033	0.0000021	0.0000044	0.0000035
*Pteropox virus*	0.0000003	0.0000003	0.0000025	0.0000009	0.0000003

* ERV* Endogenous retrovirus

### Cluster analysis of the number and relative abundance of annotated genes

**Fig. 4:**
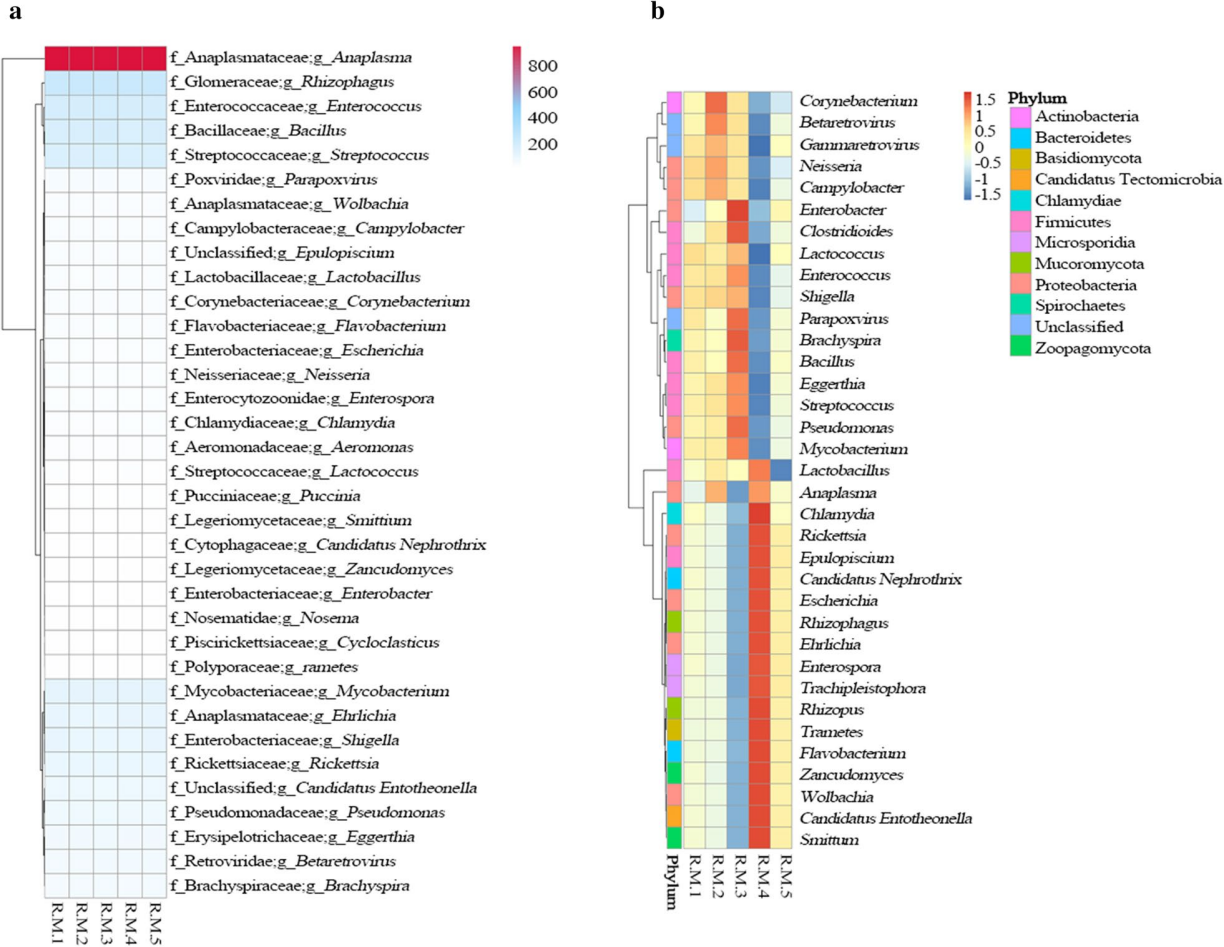
Number of genes and abundance clustering heat map at the genus level. **a** Heat map of the annotated unigene statistics. Sample names are given on the horizontal axis, and species’ information on the vertical axis;* different colors* represent the number of unigenes. **b** Relative abundance clustering heat map at the genus level. Sample information is given on the horizontal axis; the vertical axis gives the species’ information. The cluster tree on the left of the figure is the species cluster tree; the value corresponding to the middle heat map is the* Z*-value obtained after the relative abundance of each species row is standardized

The 35 most common genera and their abundance information for each sample were selected from the relative abundance tables at the different taxonomic levels to draw a heat map. The clustering was conducted at the species level to facilitate the result display and information discovery in order to identify the species clustering in the sample. The unigenes of *Anaplasma* were the most concentrated among all 35 genera in all of the samples, clustering into a single branch (Fig. 4a). The relative abundances of *Anaplasma* and *Enterobacter* were lowest in R.M.1, while the relative abundance of *Enterobacter* was high in R.M.3 (Fig. 4b).

### Gene function prediction of microflora

**Fig. 5:**
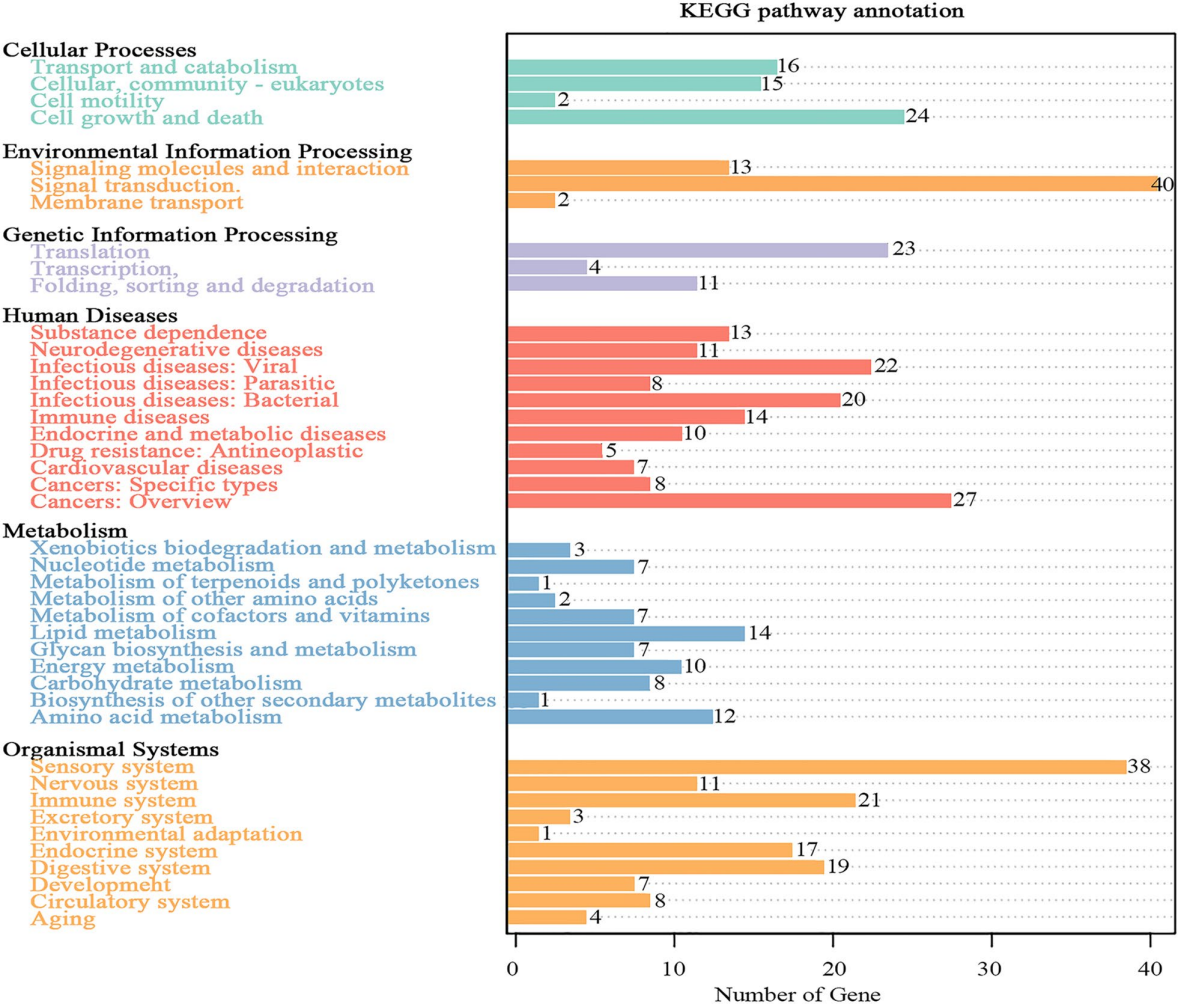
Annotations of Kyoto Encyclopedia of Genes and Genomes (KEGG) pathways related to the number of genes of the midgut microbiota in the five samples of *Rhipicephalus microplus* at level 1. The* black font* on the ordinate indicates KEGG level 1, the* colored fonts* indicate the specific pathway at this level, and the* abscissa* indicates the number of genes in the pathway

**Fig. 6:**
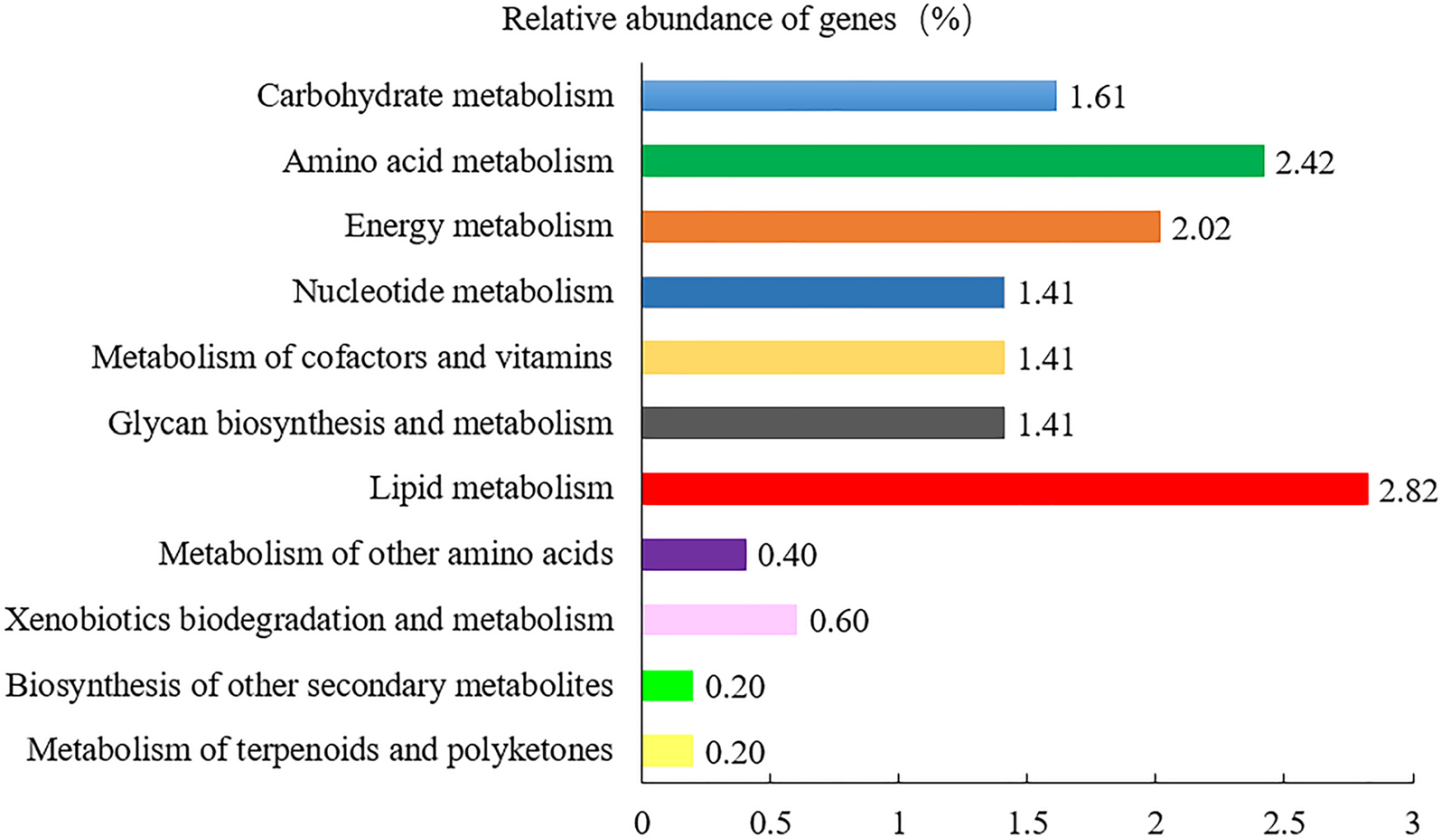
Relative abundance of midgut microbial genes involved in metabolism in the five samples of *Rhipicephalus microplus* at level 2 in KEGG pathway annotation

**Fig. 7:**
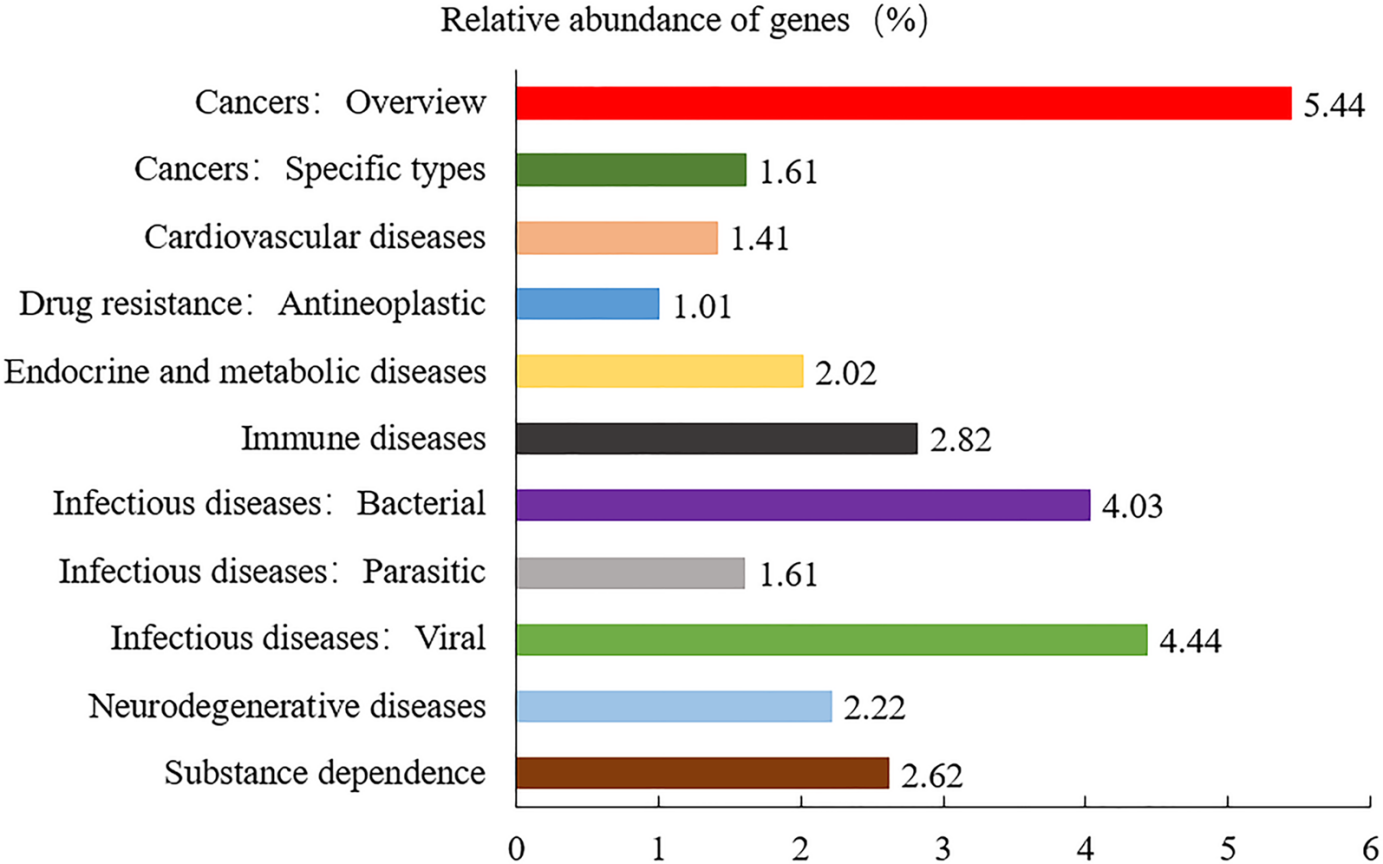
Relative abundance of midgut microbial genes involved in human diseases in the five samples of *Rhipicephalus microplus* at level 2 in KEGG pathway annotation

Unigenes were compared with the KEGG database using DIAMOND software. Based on the alignment results, the relative abundance of different functional levels was counted. At level 1, the number of KEGG pathways annotated to metabolism-related functional genes in the microbiome of *R*. *microplus* was 72; the number of functional genes associated with human diseases was 145 (Fig. 5). At level 2, genes of the microbiome of *R*. *microplus* were associated with 11 metabolic processes, among which the functional genes involved in lipid metabolism were the most abundant, followed by those involved in amino acid metabolism (Fig. 6). Genes associated with 11 human diseases, including cancer and infectious diseases (such as *Streptococcus* *pneumonia* infection, human granulocytic anaplasmosis, *Shigella* *sonnei* infection, *Salmonella enterica* infection, and pathogenic *Escherichia coli* infection), were significantly more abundant than the other functional genes (Fig. 7).

## Discussion

In this study, the microbial population in the midgut of *R*. *microplus* was investigated using a metagenomic method. Several of the main phyla, including Firmicutes, Proteobacteria, and Actinobacteria, have been reported in previous studies [55–59]. The results obtained here were somewhat consistent with those of a previous study [27] in which Proteobacteria were present at a high relative abundance. To the best of our knowledge, *Mycobacterium*, *Occidentia*, *Brachyspira*, *Campylobacter*, and *Neisseria* are reported here for the first time in *R*. *microplus*. *Coxiella* was detected in the midgut of *R*. *microplus* in a previous study [59], but it was not detected in this study. Another relevant finding of our study was the presence of *Ehrlichia*, which has previously been confirmed to exist in the midgut of *R*. *microplus* [27].

In addition, we discovered that the gene functions of the midgut microflora of *R*. *microplus* are related to lipid and amino acid metabolism; this may be due to the fact that ticks, which mostly live in agricultural areas and forests, mainly feed on the blood of their hosts [60]. The functions of some of the genes of the midgut microbiota found in the present study are associated with human diseases, and some of the most abundant microbial species are associated with infectious diseases and cancer, which suggests that ticks may be infected with various pathogens. For example, *A*. *phagocytophilum*, a tick-borne fever pathogen in ruminants [61], was detected in all of the samples.

*Anaplasma*, which belongs to the family Anaplasmataceae and is transmitted by arthropod vectors, can cause severe anemia [62]. *Anaplasma phagocytophilum* is a zoonotic pathogen found in the granulocytes of animals and humans which can infect human peripheral blood neutrophils and lead to tick-borne diseases and symptoms of human granulocytic anaplasmosis, such as fever accompanied by leukopenia, thrombocytopenia, and functional impairment [63]. Domestic animals are important hosts of *A*. *phagocytophilum* [64]. In this study, *A*. *phagocytophilum* was detected in the *R*. *microplus* samples, which were collected from cattle in Hunan province. In addition, *Ehrlichia minasensis*, which was previously detected in the hemolymph of *R*. *microplus* from Brazil [65], was also detected in this study, at low abundance. *Ehrlichia minasensis* has also recently been identified from cattle on the French island of Corsica [66], and was also found in the serum of Brazilian dogs [67].

*Occidentia*, a newly identified genus of the family Rickettsiaceae, is a Gram-negative obligate intracellular bacillus [68] that is intimately associated with its arthropod hosts [69]. *Occidentia* was isolated from the rodent-associated soft tick *Ornithodoros sonrai* collected in Senegal [68]. A recent study showed that *Occidentia* also exists in the hard tick *Africaniella transversal* [70]. In the present study, *Occidentia* was detected in the midgut of the five *R*. *microplus* examined. These findings show that *Occidentia* infects ticks of the families Argasidae and Ixodidae. Among the detected species, the endosymbionts *Rickettsia* and *Wolbachia* were also found, although at low abundances. Through their symbiosis with their host, they affect not only their host’s ecology and evolution but also its reproductive development [71]. *Wolbachia*, which is present in 66% of insect species, is probably the most abundant endosymbiont on the planet [72]. The presence of the endosymbionts *Rickettsia* in host insects, and their extensive horizontal transmission, may have contributed to their widespread occurrence in natural populations of insects [71, 73].

*Brachyspira* cause porcine intestinal spirochetosis, a condition in which diseased animals display chronic diarrhea, rectal bleeding, and lower abdominal cramps [74]. These pathogens do not cause serious disease in swine, but they do have an impact on humans. Some species of *Brachyspira*, such as *Brachyspira pilosicoli* and *Brachyspira aalborgi*, can cause similar symptoms in humans to those seen in swine [74]. *Brachyspira* have been isolated from the gastrointestinal tracts of mammals and birds, and from habitats contaminated with feces [75]. However, to date, there have been few reports of *Brachyspira*-infected cattle developing severe disease. In the present study, *Brachyspira* was detected in *R*. *microplus* at a moderately high relative abundance, which is a another reason why preventative measures should be taken to protect cattle from this tick.

The phylum Microsporidia comprises single-celled eukaryotic obligate intracellular parasites that can infect insects (e.g. *Nosema apis* and *Nosema ceranae*), fish, mammals, and even humans with immune deficiency diseases [76]. When microsporidia infect humans [77], they can cause diarrhea, myositis, keratitis, bronchitis, and encephalitis [78]. The microsporidian *S. culicis* is widely distributed and has been reported to infect Culicidae, Chironomidae, and Simuliidae [79]. In the present study, *S. culicis* was detected in the midgut of *R*. *microplus*, suggesting that this tick can carry this microsporidian, although further research is needed to determine whether *R*. *microplus* can transmit it.

Orf virus is a highly epitheliotropic parapoxvirus. It may not only cause great production losses in animal husbandry, but also affect human health [80, 81]. The clinical symptoms of orf virus infection in animals are erythema, papules, and blisters on the lips and tongue, followed by severe ulceration and, finally, the formation of scabs [82]. The pathological features of orf virus infection in humans and animals are similar and are confined to the epidermis [83]. The main clinical manifestations in humans are skin lesions on the fingers and hands after contact with infected animals [84, 85]. In addition to direct transmission, orf virus can also be transmitted by flies and ticks. The present study showed that orf virus exists in the midgut of *R. microplus*. Therefore, the eradication of *R. microplus* should be pursued in Hunan province, and orf virus monitored to ensure successful livestock farming and healthy animals.

HERV, which originate from exogenous retrovirus infections in germ cells that occurred millions of years ago, have the potential to cause human disease [86]. There are reports of increased expression of HERV-W in cases of schizophrenia and bipolar disorder, which is associated with distinct clinical or biological characteristics and symptoms [87–89]. There have been no previous reports of HERV-W in ticks. In this study, HERV were detected in the midgut of *R. microplus*, suggesting that *R. microplus* can carry these viruses.

## Conclusions

In this study, we analyzed the midgut microbiome of fully engorged adult female *R*. *microplus* from cattle in the city of Changsha in Hunan province, China, using a metagenomic sequencing method. We found that 16 phyla, 95 genera, and 144 species were common to the five samples. The midgut microbiome of this species of tick was not only composed of a large number of bacteria, but also eukaryotes and viruses. These results add to our understanding of the midgut microbiome of *R*. *microplus*. The annotated KEGG pathway predictions of the functions of the genes of the midgut microflora of *R*. *microplus* indicated that they play a role in lipid and amino acid metabolism, infectious diseases, and cancer. These findings provide fundamental information on the physiology of ticks and their transmission of disease.

## Supplementary Information

**Additional file 1: Table S1.** Relative abundance of microflora at the genus level of the five sampled ticks. **Table S2.** Relative abundance of the bacterial species of the five tick samples with the exception of the 35 most abundant species.

## Data Availability

The raw Illumina sequencing data generated in this study are available from NCBI Sequence Read Archive, BioProject no. PRJNA748905 with BioSamples accession nos. SRR15276227, SRR15276228, SRR15276229, SRR15276230 and SRR15276231.

## Abbreviations

HERV: Human endogenous retrovirus
KEGG: Kyoto encyclopedia of genes and genomes
PCR: Polymerase chain reaction
