# Supplementary material for: Metagenomics of the midgut microbiome of Rhipicephalus microplus from China
Source: Parasit Vectors. 2022 Feb 8;15:48. doi: 10.1186/s13071-022-05161-6 (PMC8822867; doi:10.1186/s13071-022-05161-6)
Supplement: Supplementary file 1 — Additional file 1: Table S1. Relative abundance of microflora at the genus level of the five sampled ticks. Table S2. Relative abundance of the bacterial species of the five tick samples with the exception of the 35 most abundant species. [file 13071_2022_5161_MOESM1_ESM.doc]

Table S1. Relative abundance of microflora of five samples at genus level.

|  | Genus | Abundance | | | | |
| --- | --- | --- | --- | --- | --- | --- |
|  |  | R.M.1 | R.M.2 | R.M.3 | R.M.4 | R.M.5 |
| Bacteria | *Streptococcus* | 0.1524515 | 0.1546662 | 0.1615159 | 0.1334146 | 0.1469918 |
|  | *Mycobacterium* | 0.1181299 | 0.1184952 | 0.1255946 | 0.1030593 | 0.1127705 |
|  | *Anaplasma* | 0.0969749 | 0.0982932 | 0.0960091 | 0.0984723 | 0.0972878 |
|  | *Enterococcus* | 0.0759854 | 0.0762666 | 0.0795191 | 0.0659992 | 0.0716191 |
|  | *Shigella* | 0.0427119 | 0.0430214 | 0.0437496 | 0.0366385 | 0.0399201 |
|  | *Lactobacillus* | 0.0257524 | 0.0263915 | 0.0258997 | 0.0276078 | 0.0236422 |
|  | *Brachyspira* | 0.0224696 | 0.0221368 | 0.0246446 | 0.0197008 | 0.0218483 |
|  | *Pseudomonas* | 0.0183686 | 0.0185518 | 0.0198552 | 0.0162925 | 0.0176557 |
|  | *Enterobacter* | 0.0168716 | 0.0175321 | 0.0193554 | 0.0162633 | 0.0177204 |
|  | *Bacillus* | 0.0127747 | 0.0124995 | 0.0139502 | 0.0108383 | 0.0123829 |
|  | *Lactococcus* | 0.0125082 | 0.0123325 | 0.0126887 | 0.0109034 | 0.0121182 |
|  | *Campylobacter* | 0.0084139 | 0.0085832 | 0.0083415 | 0.0073301 | 0.0079475 |
|  | *Rickettsia* | 0.0036892 | 0.0032390 | 0.0007805 | 0.0083605 | 0.0050558 |
|  | *Corynebacterium* | 0.0059176 | 0.0065282 | 0.0060945 | 0.0052200 | 0.0055256 |
|  | *Escherichia* | 0.0030110 | 0.0026547 | 0.0008087 | 0.0064927 | 0.0039222 |
|  | *Neisseria* | 0.0062392 | 0.0064055 | 0.0061767 | 0.0052587 | 0.0056725 |
|  | *Ehrlichia* | 0.0019209 | 0.0016806 | 0.0003970 | 0.0043562 | 0.0025865 |
|  | *Candidatus* Nephrothrix | 0.0013131 | 0.0011206 | 0.0002492 | 0.0028623 | 0.0017245 |
|  | *Wolbachia* | 0.0008269 | 0.0007424 | 0.0001854 | 0.0019280 | 0.0011074 |
|  | *Eggerthia* | 0.0015535 | 0.0015911 | 0.0016787 | 0.0013054 | 0.0014979 |
|  | *Clostridioides* | 0.0011013 | 0.0011726 | 0.0012640 | 0.0010050 | 0.0010989 |
|  | *Candidatus* Entotheonella | 0.0005071 | 0.0004457 | 0.0001062 | 0.0011905 | 0.0006818 |
|  | *Epulopiscium* | 0.0002344 | 0.0001996 | 0.0000528 | 0.0005201 | 0.0003177 |
|  | *Chlamydia* | 0.0003059 | 0.0002858 | 0.0002313 | 0.0004196 | 0.0003072 |
|  | *Flavobacterium* | 0.0001648 | 0.0001505 | 0.0000356 | 0.0004114 | 0.0002374 |
|  | *Aeromonas* | 0.0001057 | 0.0000763 | 0.0000225 | 0.0002276 | 0.0001249 |
|  | *Cycloclasticus* | 0.0000801 | 0.0000676 | 0.0000156 | 0.0001652 | 0.0000973 |
|  | *Atopobium* | 0.0001238 | 0.0000008 | 0.0001539 | 0.0001262 | 0.0001454 |
|  | *Candidatus* Regiella | 0.0000579 | 0.0000485 | 0.0000110 | 0.0001316 | 0.0000871 |
|  | *Aphanizomenon* | 0.0000517 | 0.0000375 | 0.0000090 | 0.0001166 | 0.0000700 |
|  | *Acinetobacter* | 0.0000538 | 0.0000499 | 0.0000254 | 0.0001065 | 0.0000715 |
|  | *Occidentia* | 0.0000295 | 0.0000642 | 0.0000170 | 0.0001044 | 0.0000363 |
|  | *Oceanospirillum* | 0.0000306 | 0.0000631 | 0.0000447 | 0.0000357 | 0.0000307 |
|  | *Tamlana* | 0.0000324 | 0.0000289 | 0.0000398 | 0.0000519 | 0.0000365 |
|  | *Klebsiella* | 0.0000278 | 0.0000254 | 0.0000346 | 0.0000318 | 0.0000387 |
|  | *Herbaspirillum* | 0.0000200 | 0.0000160 | 0.0000264 | 0.0000174 | 0.0000163 |
|  | *Candidatus Pelagibacter* | 0.0000200 | 0.0000167 | 0.0000163 | 0.0000138 | 0.0000194 |
|  | *Marinomonas* | 0.0000123 | 0.0000108 | 0.0000137 | 0.0000088 | 0.0000071 |
|  | *Pectobacterium* | 0.0000084 | 0.0000077 | 0.0000137 | 0.0000029 | 0.0000108 |
|  | *Paeniglutamicibacter* | 0.0000112 | 0.0000111 | 0.0000108 | 0.0000027 | 0.0000088 |
|  | *Candidatus* Magnetoglobus | 0.0000022 | 0.0000051 | 0.0000003 | 0.0000095 | 0.0000063 |
|  | *Pelotomaculum* | 0.0000044 | 0.0000060 | 0.0000052 | 0.0000026 | 0.0000016 |
|  | *Vibrio* | 0.0000014 | 0.0000049 | 0.0000003 | 0.0000054 | 0.0000015 |
|  | *Salmonella* | 0.0000020 | 0.0000009 | 0.0000054 | 0.0000032 | 0.0000010 |
|  | *Planktothrix* | 0.0000030 | 0.0000012 | 0.0000015 | 0.0000018 | 0.0000025 |
| Eukaryotes | *Rhizophagus* | 0.0061110 | 0.0054017 | 0.0013369 | 0.0137931 | 0.0082716 |
|  | *Smittium* | 0.0003336 | 0.0002908 | 0.0000676 | 0.0007816 | 0.0004510 |
|  | *Enterospora* | 0.0003404 | 0.0002874 | 0.0000645 | 0.0007332 | 0.0004577 |
|  | *Zancudomyces* | 0.0002985 | 0.0002536 | 0.0000634 | 0.0006993 | 0.0004066 |
|  | *Trametes* | 0.0001519 | 0.0001520 | 0.0000346 | 0.0003769 | 0.0002184 |
|  | *Trachipleistophora* | 0.0001442 | 0.0001312 | 0.0000301 | 0.0003238 | 0.0002033 |
|  | *Rhizopus* | 0.0001072 | 0.0001036 | 0.0000210 | 0.0002627 | 0.0001507 |
|  | *Puccinia* | 0.0001252 | 0.0000921 | 0.0000209 | 0.0002404 | 0.0001361 |
|  | *Lichtheimia* | 0.0001070 | 0.0000870 | 0.0000213 | 0.0002285 | 0.0001361 |
|  | *Armillaria* | 0.0000963 | 0.0000870 | 0.0000188 | 0.0002133 | 0.0001240 |
|  | *Sporothrix* | 0.0000951 | 0.0000759 | 0.0000184 | 0.0002106 | 0.0001198 |
|  | *Rhizoctonia* | 0.0000371 | 0.0000317 | 0.0000071 | 0.0000882 | 0.0000512 |
|  | *Nosema* | 0.0000346 | 0.0000273 | 0.0000072 | 0.0000799 | 0.0000359 |
|  | *Penicillium* | 0.0000356 | 0.0000297 | 0.0000080 | 0.0000711 | 0.0000426 |
|  | *Ceraceosorus* | 0.0000262 | 0.0000209 | 0.0000060 | 0.0000659 | 0.0000335 |
|  | *Erysiphe* | 0.0000342 | 0.0000254 | 0.0000061 | 0.0000657 | 0.0000417 |
|  | *Umbilicaria* | 0.0000200 | 0.0000177 | 0.0000040 | 0.0000524 | 0.0000227 |
|  | *Trichosporon* | 0.0000175 | 0.0000181 | 0.0000026 | 0.0000464 | 0.0000277 |
|  | *Phycomyces* | 0.0000290 | 0.0000255 | 0.0000048 | 0.0000458 | 0.0000330 |
|  | *Candida* | 0.0000204 | 0.0000188 | 0.0000053 | 0.0000453 | 0.0000274 |
|  | *Aspergillus* | 0.0000181 | 0.0000200 | 0.0000045 | 0.0000418 | 0.0000268 |
|  | *Nakaseomyces* | 0.0000153 | 0.0000119 | 0.0000018 | 0.0000394 | 0.0000206 |
|  | *Mucor* | 0.0000168 | 0.0000111 | 0.0000033 | 0.0000364 | 0.0000206 |
|  | *Tuber* | 0.0000137 | 0.0000094 | 0.0000035 | 0.0000349 | 0.0000193 |
|  | *Fusarium* | 0.0000146 | 0.0000132 | 0.0000037 | 0.0000345 | 0.0000181 |
|  | *Microbotryum* | 0.0000146 | 0.0000139 | 0.0000027 | 0.0000327 | 0.0000173 |
|  | *Choanephora* | 0.0000117 | 0.0000126 | 0.0000022 | 0.0000308 | 0.0000176 |
|  | *Mycena* | 0.0000149 | 0.0000121 | 0.0000040 | 0.0000301 | 0.0000217 |
|  | *Ganoderma* | 0.0000117 | 0.0000086 | 0.0000030 | 0.0000268 | 0.0000169 |
|  | *Chaetomium* | 0.0000124 | 0.0000115 | 0.0000026 | 0.0000259 | 0.0000189 |
|  | *Pochonia* | 0.0000099 | 0.0000082 | 0.0000020 | 0.0000243 | 0.0000132 |
|  | *Kazachstania* | 0.0000101 | 0.0000078 | 0.0000015 | 0.0000231 | 0.0000106 |
|  | *Hypholoma* | 0.0000111 | 0.0000081 | 0.0000017 | 0.0000221 | 0.0000112 |
|  | *Macrophomina* | 0.0000076 | 0.0000053 | 0.0000024 | 0.0000163 | 0.0000079 |
|  | *Piromyces* | 0.0000027 | 0.0000144 | 0.0000073 | 0.0000041 | 0.0000028 |
|  | *Marinomonas* | 0.0000123 | 0.0000108 | 0.0000137 | 0.0000088 | 0.0000071 |
|  | *Sphaerobolus* | 0.0000071 | 0.0000064 | 0.0000017 | 0.0000047 | 0.0000081 |
|  | *Gonapodya* | 0.0000032 | 0.0000046 | 0.0000011 | 0.0000077 | 0.0000037 |
|  | *Basidiobolus* | 0.0000067 | 0.0000028 | 0.0000055 | 0.0000020 | 0.0000051 |
|  | *Syncephalastrum* | 0.0000059 | 0.0000034 | 0.0000048 | 0.0000022 | 0.0000048 |
|  | *Parasitella* | 0.0000019 | 0.0000012 | 0.0000000 | 0.0000056 | 0.0000028 |
|  | *Tilletia* | 0.0000027 | 0.0000031 | 0.0000002 | 0.0000047 | 0.0000026 |
|  | *Schizosaccharomyces* | 0.0000029 | 0.0000022 | 0.0000042 | 0.0000023 | 0.0000032 |
| Viruses | Parapoxvirus | 0.0085001 | 0.0082064 | 0.0090526 | 0.0074134 | 0.0081414 |
|  | Betaretrovirus | 0.0012337 | 0.0014552 | 0.0013099 | 0.0008582 | 0.0011296 |
|  | Gammaretrovirus | 0.0002480 | 0.0002529 | 0.0002484 | 0.0002212 | 0.0002422 |
|  | Bracovirus | 0.0000526 | 0.0000554 | 0.0000101 | 0.0001018 | 0.0000655 |
|  | Scutavirus | 0.0000740 | 0.0000944 | 0.0000974 | 0.0000782 | 0.0000781 |
|  | Lymphocystivirus | 0.0000370 | 0.0000271 | 0.0000062 | 0.0000687 | 0.0000459 |
|  | Proboscivirus | 0.0000098 | 0.0000101 | 0.0000106 | 0.0000080 | 0.0000076 |

Table S2. Relative abundance of other than the top 35 bacterial species of five tick samples.

| Species | Abundance | | | | |
| --- | --- | --- | --- | --- | --- |
|  | R.M.1 | R.M.2 | R.M.3 | R.M.4 | R.M.5 |
| *Entomoplasmatales bacterium* EntAcro10 | 0.0001319 | 0.0001220 | 0.0000270 | 0.0003034 | 0.0001890 |
| *Aeromonas cavernicola* | 0.0001057 | 0.0000763 | 0.0000225 | 0.0002276 | 0.0001249 |
| *Cycloclasticus* sp. symbiont | 0.0000801 | 0.0000676 | 0.0000156 | 0.0001652 | 0.0000973 |
| *Streptococcus mutans* | 0.0001454 | 0.0001257 | 0.0001612 | 0.0001075 | 0.0001246 |
| *Atopobium vaginae* | 0.0001238 | 0.0000008 | 0.0001539 | 0.0001262 | 0.0001454 |
| *Chlamydia abortus* | 0.0000661 | 0.0000518 | 0.0000221 | 0.0001417 | 0.0000854 |
| *Candidatus* Regiella insecticola | 0.0000579 | 0.0000485 | 0.0000110 | 0.0001316 | 0.0000871 |
| *Aphanizomenon flos-aquae* | 0.0000517 | 0.0000375 | 0.0000090 | 0.0001166 | 0.0000700 |
| *Acinetobacter baumannii* | 0.0000524 | 0.0000493 | 0.0000235 | 0.0001065 | 0.0000715 |
| *Occidentia massiliensis* | 0.0000295 | 0.0000642 | 0.0000170 | 0.0001044 | 0.0000363 |
| *Epulopiscium* sp. Nele67-Bin001 | 0.0000438 | 0.0000334 | 0.0000099 | 0.0001014 | 0.0000523 |
| *Bacteroidetes bacterium* 4572_77 | 0.0000305 | 0.0000225 | 0.0000059 | 0.0000682 | 0.0000395 |
| *Oceanospirillum multiglobuliferum* | 0.0000306 | 0.0000631 | 0.0000447 | 0.0000357 | 0.0000307 |
| *Tamlana* sp. s12 | 0.0000324 | 0.0000289 | 0.0000398 | 0.0000519 | 0.0000365 |
| *Chlamydia trachomatis* | 0.0000076 | 0.0000233 | 0.0000072 | 0.0000480 | 0.0000100 |
| *Klebsiella pneumoniae* | 0.0000278 | 0.0000254 | 0.0000346 | 0.0000318 | 0.0000387 |
| *Clostridiales bacterium* 1_7_47FAA | 0.0000223 | 0.0000264 | 0.0000335 | 0.0000147 | 0.0000215 |
| *Flavobacteriales bacterium* | 0.0000148 | 0.0000093 | 0.0000036 | 0.0000292 | 0.0000203 |
| *Planctomycetia bacterium* TMED53 | 0.0000150 | 0.0000075 | 0.0000011 | 0.0000283 | 0.0000156 |
| *Bacillus vireti* | 0.0000270 | 0.0000258 | 0.0000273 | 0.0000263 | 0.0000210 |
| *Herbaspirillum rubrisubalbicans* | 0.0000109 | 0.0000130 | 0.0000223 | 0.0000113 | 0.0000138 |
| *Candidatus* Pelagibacter ubique | 0.0000200 | 0.0000167 | 0.0000163 | 0.0000138 | 0.0000194 |
| *Pseudomonas stutzeri* | 0.0000093 | 0.0000068 | 0.0000019 | 0.0000192 | 0.0000118 |
| *Epulopiscium* sp. Nele67-Bin004 | 0.0000084 | 0.0000066 | 0.0000017 | 0.0000168 | 0.0000103 |
| *Pseudomonas aeruginosa* | 0.0000119 | 0.0000160 | 0.0000116 | 0.0000131 | 0.0000065 |
| *Marinomonas spartinae* | 0.0000123 | 0.0000108 | 0.0000137 | 0.0000088 | 0.0000071 |
| *Pectobacterium carotovorum* | 0.0000084 | 0.0000077 | 0.0000137 | 0.0000029 | 0.0000108 |
| *Paeniglutamicibacter antarcticus* | 0.0000112 | 0.0000111 | 0.0000108 | 0.0000027 | 0.0000088 |
| *Gammaproteobacteria bacterium* 2W06 | 0.0000079 | 0.0000103 | 0.0000089 | 0.0000050 | 0.0000080 |
| *Candidatus* Magnetoglobus multicellularis | 0.0000022 | 0.0000051 | 0.0000003 | 0.0000095 | 0.0000063 |
| *Herbaspirillum* sp. VT-16-41 | 0.0000091 | 0.0000030 | 0.0000041 | 0.0000061 | 0.0000025 |
| *Epulopiscium* sp. AS2M-Bin002 | 0.0000020 | 0.0000048 | 0.0000006 | 0.0000091 | 0.0000037 |
| *Epulopiscium* sp. SCG-B11WGA-EpuloA1 | 0.0000052 | 0.0000026 | 0.0000033 | 0.0000066 | 0.0000030 |
| *Pseudomonas pelagia* | 0.0000037 | 0.0000065 | 0.0000048 | 0.0000057 | 0.0000027 |
| *Pelotomaculum thermopropionicum* | 0.0000044 | 0.0000060 | 0.0000052 | 0.0000026 | 0.0000016 |
| *Vibrio parahaemolyticus* | 0.0000014 | 0.0000049 | 0.0000003 | 0.0000054 | 0.0000015 |
| *Salmonella enterica* | 0.0000020 | 0.0000009 | 0.0000054 | 0.0000032 | 0.0000010 |
| *Bathymodiolus platifrons* methanotrophic gill symbiont | 0.0000019 | 0.0000017 | 0.0000006 | 0.0000050 | 0.0000040 |
| *Bacillus anthracis* | 0.0000011 | 0.0000021 | 0.0000040 | 0.0000018 | 0.0000027 |
| *Planktothrix tepida* | 0.0000030 | 0.0000012 | 0.0000015 | 0.0000018 | 0.0000025 |
| *Mycobacterium szulgai* | 0.0000015 | 0.0000024 | 0.0000020 | 0.0000012 | 0.0000022 |
